# Asexualization for Crime

**Published:** 1897-06

**Authors:** 


### Abstracts and Selections.

### Asexualization for Crime.

*Leonard's Illustrated Medical Journal* gives the following bill, which has been introduced in the legislature of Michigan, and has a fair chance to become a law:

"SECTION 1. The people of the State of Michigan enact, That all persons inmates of the Michigan home for the Feeble Minded and Epileptic, and all persons who shall hereafter become inmates of said Home for the Feeble Minded and Epileptic, that each and every person confined in said institution, and before he or she is discharged, shall be caused to submit to an operation that causes asexualization, that such persons shall cease to be able to reproduce their kind.

"SEC. 2. All persons who shall have been convicted of a felony a third time, and so stated by the court, the first or second conviction having been committed in this State or some other State of the United States, upon conviction and sentence to a Michigan State prison, all of such persons so convicted and sentenced at a time prior to the expiration of such known third sentence, shall be caused to summit to an operation that causes asexualization and stops their ability to reproduce their kind.

"Sec. 3. The superintendent, warden or other person having charge of such Home for the Feeble Minded and Epileptic, and such prisons as shall contain such persons, as provided for in sections 1 and 2 of this act; the medical superintendent in charge of said institution shall perform or assist in the performance of the same any physician or surgeon of the State. The superintendent, warden or other person in charge of said institution may pay to each operator a sum not more than twenty-five dollars for each and every operation so performed; and in no case where the operation is performed by the physician regularly employed by the within named institution shall there be paid any extra compensation.

"Sec. 4. In each and every case, before such operation shall be performed, if the person be feeble minded or an epileptic confined within a prison in this State, the matter shall be presented in writing to the board of control of such institution, wherein it shall be shown that such operation would benefit the subject physically and morally, or that it is necessary as a restrictive measure to prevent propagation of any kind in case the subject is discharged from the institution. The board of control shall, after being satisfied of the advisability of such operation, authorize the medical superintendent to perform the same, after first giving notice in writing to the parents or guardians of such persons at least ten days before such operation.

"Sec. 5. That whoever shall have been convicted of the crime of having ravished a child or woman while upon the streets of any city, village, public highway or any other place within this State, it shall be the duty of the jury making such sentence to include in such sentence that within one year after being confined in such prison, an operation which causes asexualization shall be performed as provided in sections 3 and 4 of this act.

"Sec. 6. The penalty for non-compliance of this act shall be just cause for removal and forfeiture of the position of such superintendent, warden or other person named in this act."

A somewhat similar bill was introduced into the Texas Legislature four years ago, which bill was the pioneer in this line,—the measure having first been suggested and urged by the Texas Medical Journal. Since then Kansas has gotten a bill of the kind enacted into a law, and it is in operation. Were emosculation and imprisonment substituted for capital punishment, in after years we would have less rape and fewer lynchings; and if, at same time our marriage laws were properly amended, there would be fewer Guiteaus and Pendergasts.

---

**For** acute bronchitis—

℞ Ammonia muriate..................gr. 80
Tartar emetic.........................gr. 1
Iodide potas..........................gr. 16
Simple elixir .........................℥ 4
Aquæ destil...........................qs. ad ℥8

Sig.: A tablespoonful every 3 or 4 hours.

The above will afford quick relief to the congested mucous membrane of the bronchial tubes, and rapidly establish mucous secretion.

---

**A Remedy for Seasickness.**—The *Progres Medical* takes this formula of Barber's from the *Semaine Medicale:*

℞ Chloroform, Tincture of nux vomica, } each...10 drops.

Compound tincture of lavender....1 fl. drachm.

Water.....................................10 fl. drachms.

M. A teaspoonful to be taken every hour until the vomiting and nausea have subsided, care being taken to shake the bottle each time before pouring out the dose.—*New York Medical Journal.*

---

**The Bio-Chemic Springs.** Texas is remarkably rich in mineral waters, many of her richest wells and springs being unknown outside the State. Few persons out of Texas are aware that at Paris, Texas, for instance, there is a wonderful spring—to which the above name has been given, an analysis of whose waters shows a remarkable resemblance to the famous Carlsbad Springs. The analysis, made by State Geologist, E. T. Dumble, shows that this water is very rich in the carbonates and sulphates of sodium, and sulphate of magnesium and calcium, with some iron; properties which render the water very useful in a wide range of pathological state. Mr. Isaac Bernstein, the owner, will be pleased to furnish analysis free for the asking; and also to sell you the water. It deserves to be better known.

